# Real-Time CARS Imaging Reveals a Calpain-Dependent Pathway for Paranodal Myelin Retraction during High-Frequency Stimulation

**DOI:** 10.1371/journal.pone.0017176

**Published:** 2011-03-03

**Authors:** Terry B. Huff, Yunzhou Shi, Wenjing Sun, Wei Wu, Riyi Shi, Ji-Xin Cheng

**Affiliations:** 1 Department of Chemistry, Purdue University, West Lafayette, Indiana, United States of America; 2 Weldon School of Biomedical Engineering, Purdue University, West Lafayette, Indiana, United States of America; 3 Department of Basic Medical Sciences, Purdue University, West Lafayette, Indiana, United States of America; The Research Center of Neurobiology-Neurophysiology of Marseille, France

## Abstract

High-frequency electrical stimulation is becoming a promising therapy for neurological disorders, however the response of the central nervous system to stimulation remains poorly understood. The current work investigates the response of myelin to electrical stimulation by laser-scanning coherent anti-Stokes Raman scattering (CARS) imaging of myelin in live spinal tissues in real time. Paranodal myelin retraction at the nodes of Ranvier was observed during 200 Hz electrical stimulation. Retraction was seen to begin minutes after the onset of stimulation and continue for up to 10 min after stimulation was ceased, but was found to reverse after a 2 h recovery period. The myelin retraction resulted in exposure of Kv 1.2 potassium channels visualized by immunofluorescence. Accordingly, treating the stimulated tissue with a potassium channel blocker, 4-aminopyridine, led to the appearance of a shoulder peak in the compound action potential curve. Label-free CARS imaging of myelin coupled with multiphoton fluorescence imaging of immuno-labeled proteins at the nodes of Ranvier revealed that high-frequency stimulation induced paranodal myelin retraction via pathologic calcium influx into axons, calpain activation, and cytoskeleton degradation through spectrin break-down.

## Introduction

High-frequency electrical stimulation of the central nervous system (CNS) is becoming a promising therapy for treatment of a variety of disorders. Electrical stimulation of the brain has provided new treatment options for Parkinson's disease and epilepsy [1], [2] whereas stimulation of the spinal cord shows potential for chronic pain management [3], [4]. However, the response of the CNS to extended high-frequency stimulation is not well understood [5], [6].

It was previously reported that repetitive activity resulted in changes in the magnitude, duration, and repolarization rate of compound action potentials (CAPs) in peripheral nerve tissue [7]. It was further suggested that high-frequency stimulation could loosen the paranodal axoglial junctions thereby impacting the action potential [8]. Structural deformation of myelin following repetitive CAP propagation has been visualized by histology [9], X-ray diffraction [10], and electron microscopy [11], [12]. Nevertheless, real-time visualization of structural changes of paranodal myelin upon repetitive stimulation was impossible with these traditional techniques. Consequently, the mechanism linking electrical stimulation and paranodal myelin deformation has not been clarified.

Nonlinear optical microscopy is becoming a powerful tool in neuroscience [13]. Two-photon excited fluorescence (TPEF) microscopy [14], [15] and sum frequency generation microscopy [16] have been applied to visualize cellular activities in brain and astroglial filaments in spinal cord, respectively. Coherent anti-Stokes Raman scattering (CARS) microscopy, which possesses vibrational selectivity, has received additional attention recently [17], [18], [19] and has been demonstrated to be particularly sensitive to lipid rich structures based on the resonant CARS signal from C-H vibration [20], [21], [22]. The high lipid-to-protein ratio of myelin makes CARS a sensitive imaging tool for this structure [23]. Although the myelin sheath can be labeled in live tissues by lipophilic dyes [24], [25], CARS microscopy has allowed label-free visualization of the myelin sheath in sciatic nerves in live mice [26] and rats [27], and has further been employed in a mechanistic study of demyelination induced by lysophosphatidylcholine [28] and glutamate excitotoxicty [29] in live spinal cord white matter tissues.

In this paper we apply real-time CARS imaging to monitor changes to the paranodal myelin of central myelinated nerve fibers during high-frequency stimulation. We observed paranodal myelin retraction in isolated spinal cord white matter strips during high-frequency stimulation and quantified the extent of myelin retraction via label-free examination of a large number of nodes, which helped to elucidate the mechanisms linking myelin retraction and repetitive simulation. Moreover, we have combined CARS imaging with CAP recording to correlate the myelin structural change with the collective electrophysiological response.

## Materials and Methods

### Preparation of live spinal tissue

Fresh spinal cord white matter strips were isolated from guinea pigs [30]. Briefly, adult guinea pigs were deeply anesthetized by injection of ketamine/acepromazine/xylazine (80 mg/kg ketamine, 0.8 mg/kg acepromazine, 12 mg/kg xylazine, i.p.), followed by cardiac perfusion with cold, oxygenated Krebs' solution (NaCl 124 mM, KCl 2 mM, KH_2_PO_4_ 1.2 mM, MgSO_4_ 1.3 mM, CaCl_2_ 2 mM, dextrose 10 mM, NaHCO_3_ 26 mM, and sodium ascorbate 10 mM). The whole spinal cord was then removed and split by mid-line sagittal division. The sections were then cut radially to separate the ventral white matter from the grey matter. Isolated ventral white matter strips with a length of about 4 cm were maintained in Krebs' solution (bubbled with 95% O_2_ and 5% CO_2_) for 1 h prior to experiments. All procedures were approved by the Purdue Animal Care and Use Committee (protocol 05-045).

### Coupling of CAP recording with CARS imaging

CAP was measured using a double sucrose gap chamber [30] which was specially made with a glass coverslip bottom to facilitate simultaneous CARS imaging of the same sample. The white matter strip was draped across the electrodes in the two end chambers and allowed to rest on the glass coverslip bottom of the center chamber. The two end chambers were filled with 120 mM KCl. The Krebs' solution enriched with oxygen was maintained in a 37°C water bath and was circulated through the center chamber at a flow rate of 2 mL/min.

**Figure 1:**
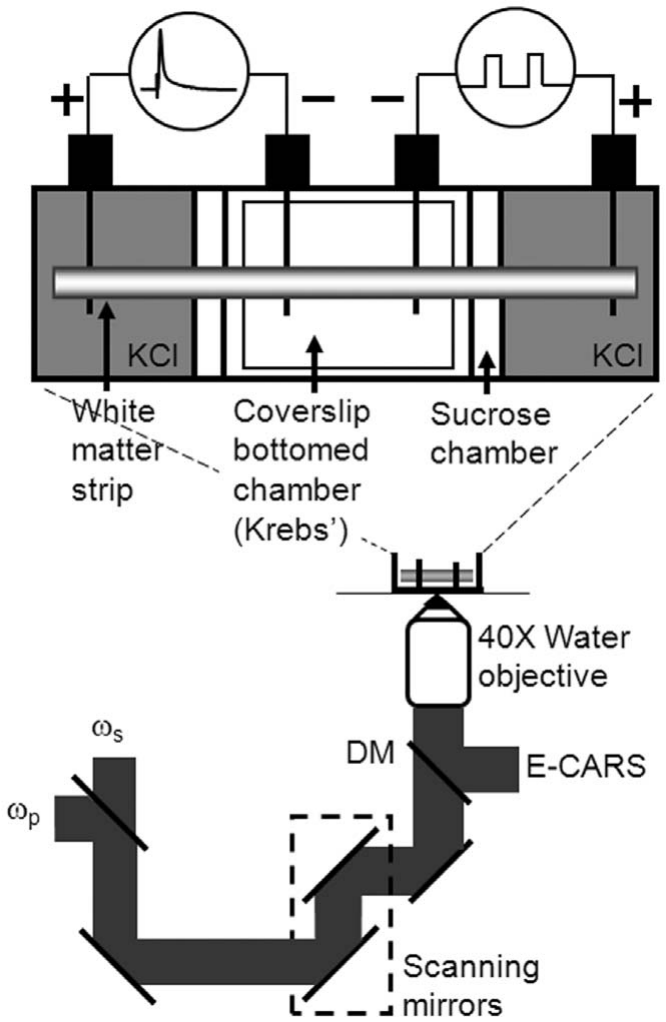
Experimental setup for simultaneous E-CARS imaging, high-frequency stimulation, and CAP recording. DM: dichroic mirror.

To facilitate real-time imaging of paranodal myelin dynamics during high-frequency stimulation, the chambered unit was placed on an inverted laser-scanning microscope (Olympus IX70/FV300). A diagram of the experiment setup is shown in Fig. 1. The CARS signal was generated by two Ti:sapphire oscillators (Mira 900, Coherent, Santa Clara, CA) tightly synchronized (Sync-Lock, Coherent) and collinearly combined using a dichroic combiner (LWP-45-R720-7850-PW-1004-UV, CVI Laser LLC, Albuquerque, NM). The frequency difference of the pump (705.6 nm) and stokes (882.5 nm) lasers were tuned to 2840 cm^−1^, the peak of the symmetric CH_2_ stretching vibration. The combined beams were passed through a Pockels' cell (Model 350-160, Conoptics, Danbury, CT) to reduce the repetition rate to between 1–4 MHz in order to prevent photodamage to the spinal tissue [31]. The beams were focused into the sample by a 40× objective (LUM PlanFl/IR, numerical aperture (N.A.) = 0.8, Olympus) or 60× objective (UPlanApo/IR, NA = 1.2, Olympus). The epi-detected CARS (E-CARS) signal was then collected by the objective, separated from the excitation light by a dichroic mirror (670 dcxr, Chroma Technology Corp., Rockingham, VT), spectrally filtered (42-7336, 600/65 nm, Ealing Catalog Inc., Rocklin, CA), and detected by an external photomultiplier tube (PMT, H7422-40, Hamamatsu, Japan) mounted at the back-port of the microscope.

Prior to the start of experiments, the samples were stimulated with 0.1 ms pulses at a frequency of 1 Hz until the CAP had stabilized. The electrode potential used for each sample was determined as twice the minimal potential necessary to elicit the maximum observed CAP amplitude. To monitor myelin dynamics during high-frequency stimulation, a series of real-time E-CARS images were acquired at a rate of one frame per second at an interval of 20 seconds during 200 Hz stimulation and for 45 min post stimulation.

### Calcium labeling and imaging

Ventral white matter strips were incubated for 1 h in Ca^2+^ free Krebs' solution (bubbled with 95% O_2_ and 5% CO_2_) containing 2 mM MgCl_2_ and 40 µM Oregon Green BAPTA2-AM (OG, Invitrogen), a cell permeant calcium indicator. Samples were then transferred to the double sucrose gap chamber and normal Krebs' solution was circulated through the center chamber at 2 mL/min. The samples were stimulated at 1 Hz until the CAP was stable after which samples were stimulated at 200 Hz for 15 min. After stimulation, samples were examined by E-CARS imaging of myelin sheath and TPEF imaging of OG.

### Immunofluorescence

White matter strips were labeled with antibodies against Kv 1.2 (Chemicon, Temecula, CA), contactin-associated protein (Caspr), AnkyrinB, NFC2 (Antibodies Inc., CA) or βII spectrin (BD Bioscience, USA) according to established protocols [32], [33]. Briefly, 1-cm segments of samples stimulated at 1 Hz for 15 min as well as those stimulated at 200 Hz for 15 min were fixed in 4% paraformaldehyde solution for 1 h for βII spectrin staining or 24 h otherwise. Then samples stained for Kv 1.2, Caspr, AnkyrinB, NFC2 were sectioned into 50 µm thick slices and samples for anti-βII spectrin labeling were sectioned into 100 µm with a vibratome (OT-4000 Electron Microscope Sciences). The slices were incubated in 3% Triton X-100 in PBS for 30 min, followed by phosphate buffered saline (PBS) containing 10% goat serum and 0.3% Triton X-100 for 1 h, and then washed in PBS for 1 h. The samples were then incubated in primary antibodies dissolved in PBS containing 1% goat serum and 0.1% triton X-100 at a concentration of 1∶100 for 24 h at 4°C and washed 3 times for 30 min. The slices were then treated with FITC-goat anti-rabbit IgG (H+L) (Invitrogen), FITC-goat anti-mouse IgG (H+L) or Cy3 anti-mouse IgG (H+L) (Sigma), respectively, as the secondary antibody in PBS containing 1% goat serum and 0.1% triton X-100 (1∶100) for 2 h. The samples were then washed 3 times for 30 min. The samples were examined by simultaneous forward-detected CARS (F-CARS) imaging of myelin and epi-detected TPEF imaging of FITC conjugated secondary antibody. The TPEF signal was spectrally filtered (HQ520-40, 520/40 nm, Ealing) prior to detection by PMT. Samples with double labeling were imaged by a confocal fluorescence microscope (Olympus), with FITC signal excited by 488 nm laser and Cy3 signal excited by 543 nm laser.

### Statistical analysis

The percentage of myelinated nodes was counted for each cord and averaged within the group. Significance between groups was determined by One-way ANOVA using the Tukey test.

## Results

**Figure 2:**
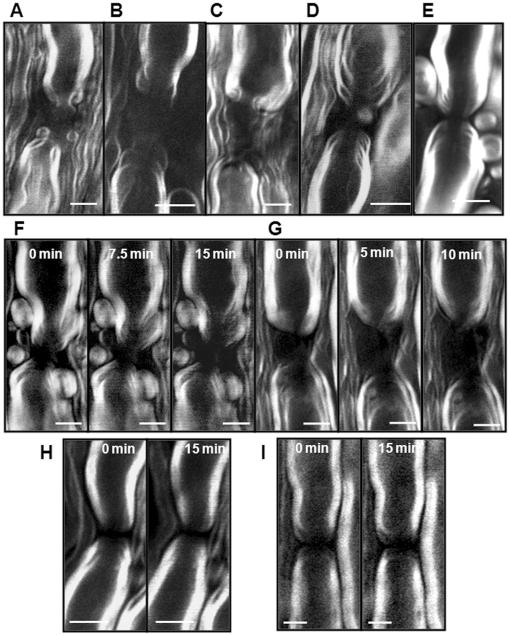
Myelin retraction following high-frequency stimulation. (A–D) Myelin retraction and splitting is clearly seen at nodes of Ranvier after high-frequency stimulation. (E) Myelin morphology appears normal in non-stimulated controls. (F) Real-time E-CARS imaging of paranodal myelin retraction during stimulation at 200 Hz. Left: 0 min, middle: 7.5 min, right: 15 min. (G) Myelin retraction continues after stimulation is ceased. Left: immediately after stimulation, middle: 5 min post-stimulation, right: 10 min post-stimulation. After a period of additional drawback, no further myelin changes are observed. (H) E-CARS images of node of Ranvier in spinal tissue maintained in circulating normal Krebs' for 15 min show no myelin retraction during continuous imaging. Images acquired at an interval of 20 seconds for 15 minutes with each image acquired in 1 second. (I) E-CARS images of node of Ranvier in spinal tissue sample stimulated at 1 Hz for 15 min show no myelin retraction. Bar = 5 µm in all images.

With the resonant signal from the CH_2_ vibration band, CARS microscopy allowed label-free imaging of paranodal myelin structure as shown in Fig. 2. After stimulation of samples at 200 Hz for 15 min, retraction of paranodal myelin from the node of Ranvier was extensively observed (Fig. 2A–D). In contrast, myelin morphology at the nodes of Ranvier from non-stimulated samples maintained in circulating Krebs' solution appeared healthy (Fig. 2E). Real-time E-CARS imaging showed that retraction began shortly after the onset of stimulation and continued throughout the duration of stimulation (Fig. 2F and Supplemental Movie S1). During stimulation the distance between the paranodal bulbs was seen to increase by several microns and there did not appear to be any clear preference for either proximal or distal paranodal bulbs as retraction was seen in both. In some instances, myelin retraction could still be observed to continue for up to 10 min after stimulation was ceased (Fig. 2G). After this period of continued drawback, no further changes to paranodal myelin structure were observed for up to 45 min post-stimulation. To verify that the observed myelin retraction and splitting was due to repetitive stimulation, control samples were imaged continuously at a rate of one frame per second and an interval of 20 s for 15 min without stimulation (Fig. 2H). No retraction of paranodal myelin was observed. Additionally, samples stimulated at frequency of 1 Hz for 15 min showed no retraction (Fig. 2I).

**Figure 3:**
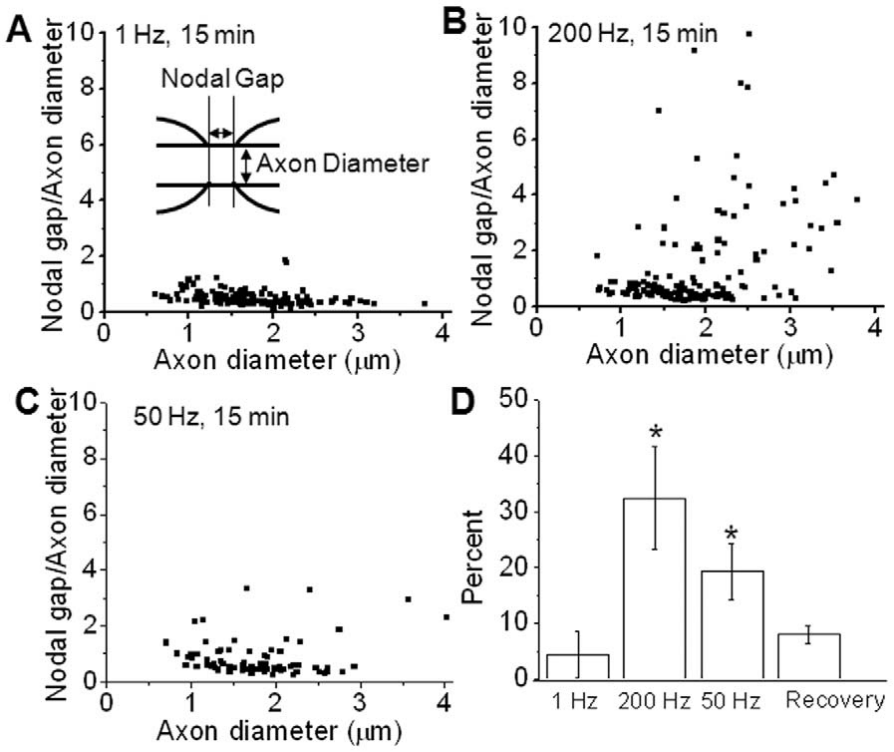
Dependence of paranodal myelin retraction on stimulation frequency. (A) Inset: Diagram of node of Ranvier defining parameters measured. Plot: Scatter-plot of ratio of Nodal Gap/Axon Diameter versus Axon Diameter for spinal tissue samples stimulated at 1 Hz for 15 min (8 samples, 144 nodes total) (B) Scatter-plot of ratio of Nodal Gap/Axon Diameter vs. Axon Diameter for samples stimulated at 200 Hz for 15 min (4 samples, 150 nodes total). Plot shows that at 200 Hz, larger axons are more susceptible to retraction evidenced by the increased ratio for larger axon diameters. (C) Scatter-plot of ratio of Nodal Gap/Axon Diameter vs. Axon Diameter for samples stimulated at 50 Hz for 15 min in normal Krebs' (n = 3, 76 nodes total). (D) Bar graph of percentage of nodes showing retracted morphology. Samples stimulated at 1 Hz, 200 Hz, and 50 Hz for 15 min show 4±4%, 32±9%, and 19±5% of nodes with retracted morphology, respectively. After a 2 h recovery period, myelin retraction was reduced to 8±2%. * indicates data with significant difference (p<0.01, One-way ANOVA).

To quantitatively correlate the extent of paranodal myelin retraction with stimulation frequencies, E-CARS images were acquired of white matter strips separated into 3 groups: 1 Hz stimulation (8 strips), 200 Hz stimulation (4 strips), and 50 Hz stimulation (3 strips). Each white matter strip in normal Krebs' underwent the stated stimulation protocol for 15 min and nodes were measured by E-CARS imaging immediately after the stimulation was ceased. The sum total of nodes measured from all strips in each group were 144 (1 Hz), 150 (200 Hz), and 76 (50 Hz). The sizes of axons studied were within the typical size distribution for guinea pig ventral axons [34]. From these images, we measured the axon diameter at the paranode as well as the nodal gap, defined as the distance between the paranodal bulbs. Because CARS lacks the sensitivity to visualize the axon membrane, axon diameters were determined by measuring the distance between the paranodal loops across a single paranodal bulb (Fig. 3A inset). The ratio of the nodal gap to axon diameter was used to provide a quantitative measurement of myelin retraction under different stimulation conditions (Fig. 3A–C). It was observed that in samples stimulated at 1 Hz this ratio was typically less than 1.0 regardless of axon size (Fig. 3A). Therefore “healthy” nodes were defined as those with a ratio less than 1.0 whereas nodes with retracted myelin were defined as those which possessed a ratio greater than 1.0. In control samples, 4±4% of nodes showed retracted paranodal myelin (Fig. 3D). This small percentage of retracted nodes could be attributed to mechanical stress from sample handling during extraction and sectioning of the spinal tissues. Stimulation at 200 Hz resulted in significantly greater number of myelin-retracted nodes (32±9%, Fig. 3D), with an increase of the nodal gap to axon diameter ratio up to as high as 9.8∶1 (Fig. 3B). Paranodal myelin retraction demonstrated a clear dependence on stimulation frequency as 50 Hz stimulation for 15 min (Fig. 3C) resulted in 19±5% myelin retraction (Fig. 3D). Statistical comparison by One-way ANOVA of the number of myelin retracted nodes showed a significant difference between each of the stimulation parameters (p<0.01). Notably, the myelin retraction percentage also showed a strong dependence on the axon diameter. For 200 Hz stimulation, the myelin retraction percentages from all nodes examined were 11%, 16%, 44%, and 85% for axons with diameter *d* within 0<d≤1, 1<d≤2, 2<d≤3, and 3<d≤4 µm, respectively.

In order to evaluate the potential reversibility of paranodal myelin retraction, four white matter strips were stimulated at 200 Hz for 15 min and were then maintained in Krebs' solution for 2 h. Afterwards, myelin retracted nodes were quantified by E-CARS imaging (88 nodes total). After a 2 h recovery period, myelin retraction was reduced to 8±2% (Fig. 3D). Statistical analysis by One-way ANOVA showed no significant difference (p>0.1) between recovered samples and controls stimulated at 1 Hz, but did show a significant difference (p<0.01) between recovered samples and those imaged immediately after either 200 Hz or 50 Hz stimulation (Fig. 3D).

**Figure 4:**
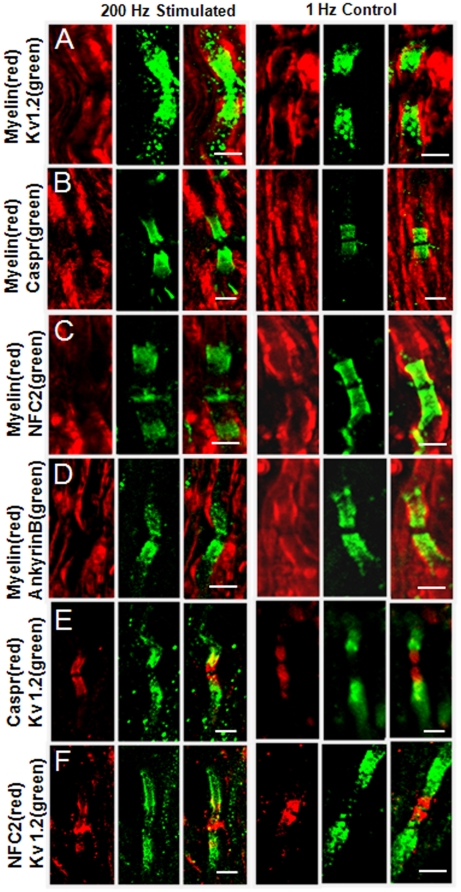
Distribution of paranodal junction and cytoskeleton proteins after 200-Hz stimulation compared with the 1-Hz stimulation group. (A) Kv 1.2 diffuses into the nodal area. (B) Caspr remained in the paranodes. (C) NF155 elongated and extended beyond paranodes while NF 186 retained. Both NF155 and NF 186 were labeled by the NFC2 antibody. (D) AnkyrinB remained in paranodes. (E) Partial overlap of Kv 1.2 and Caspr after 200 Hz stimulation (Fig. 4E) confirms the diffusion of Kv1.2 beyond the juxtaparanodal area. (F) Partial overlap of immuno-labeled NFC2 and Kv 1.2 confirms their redistribution. Bar = 5 µm for all images.

Paranodal myelin retraction as observed by CARS may be associated with exposure of juxtaparanodal K^+^ channels. Because fast-responding voltage gated potassium channels such as the 1.2 type lie beneath the juxtaparanodal myelin, immunolabeling of white matter strips with a Kv 1.2 antibody was applied to samples stimulated at 200 Hz as well as controls that were stimulated at 1 Hz for the same amount of time. By TPEF imaging of anti-Kv1.2 and E-CARS imaging of myelin, control samples showed that Kv 1.2 channels located beneath the myelin at the juxtaparanode. In contrast, samples stimulated at 200 Hz showed myelin retraction at the nodes of Ranvier with redistributed Kv 1.2 channels (Fig. 4A). To correlate myelin retraction with Kv 1.2 channel exposure, nodes of Ranvier (40 nodes total) were examined from 3 separate spinal cord strips that were stimulated at 200 Hz followed by Kv 1.2 immunolabeling. 33±4% of nodes examined showed myelin retraction. Additionally all of these nodes showed potassium channel exposure whereas none of the nodes which possessed normal paranodal myelin morphology showed Kv 1.2 channel exposure. We characterized the localization of Kv 1.2 before stimulation, after stimulation and after a two hour recovery period by measuring the distance between Kv 1.2 on each side of the node normalized by axon diameter (Fig. S1). After 200 Hz stimulation, the distance ratio was reduced to 0.59±0.14 from 2.15±0.25 (*p*<0.01). We further investigated several other nodal proteins that are responsible for axon-myelin association [32]. The junction protein Caspr (Fig. 4B) relocated further away from the paranodal area and diffused along the axon. we quantified the location of Caspr by measuring the gap distance between Caspr at each side of the node normalized by the axon diameter (supplementary figure S2). The nodal gap ratio increased to 1.49±0.17 in 200 Hz stimulated spinal cords as compared with 0.53±0.05 in 1 Hz control (*p*<0.01). We also characterized the length of Caspr by measuring the length of the fluorescence labels normalized by the axon diameter. The Caspr length ratio increased to 5.64±0.94 in 200 Hz stimulated spinal cords while the control showed 3.97±0.32 (*p*<0.01) (Fig. S1). Similarly, anti-NFC2 which labels both neurofascin 155 (NF 155) proteins at paranodes and neurofascin 186 (NF 186) proteins at nodes (Fig. 4C) diffused from paranodes to juxtaparanodes. The cytoskeleton protein AnkyrinB appeared to remain intact after myelin retraction (Fig. 4D). Co-staining of Kv 1.2 and Caspr showed some overlap of these two proteins after 200 Hz stimulation (Fig. 4E), which confirms the diffusion of Kv1.2 beyond the juxtaparanodal area. Moreover, overlap of immno-labeled NFC2 and Kv 1.2 (Fig. 4F) confirms their redistribution (c.f. Fig. 4A, C) under the high-frequency stimulation condition.

**Figure 5:**
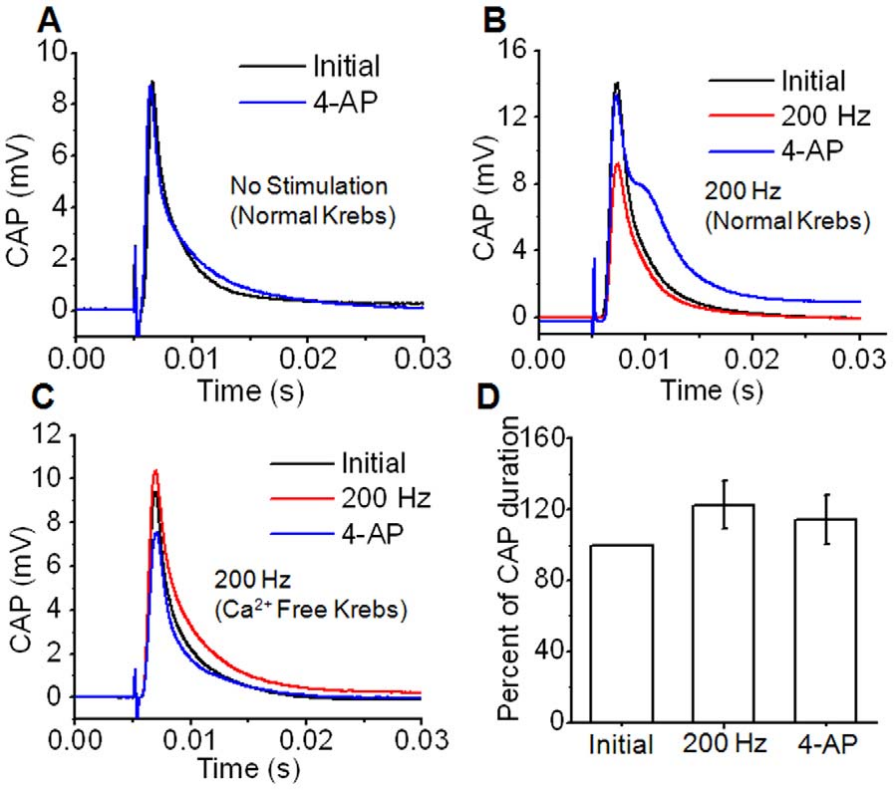
Compound action potential measurement and treatment with 4-aminopyradine (4-AP) demonstrates Ca^2+^ dependence. (A) Control sample maintained in normal Krebs' shows no clear change to CAP waveform after 4-AP treatment. (B) After 200 Hz stimulation, 4-AP treatment results in the appearance of a shoulder in the CAP. (C) Samples stimulated at 200 Hz in Ca^2+^ free Krebs' show significantly reduced broadening and no additional peaks. (D) For the same samples in (C), statistical comparison before stimulation, after stimulation, and after 4-AP treatment shows no significant increase to CAP duration (n = 5, p>0.1, ANOVA).

The CARS visualization of myelin retraction and immunofluorescence observation of Kv 1.2 channel exposure was further verified by an electrophysiological study. A potassium channel blocker, 4-aminopyridine (4-AP), has previously been demonstrated to broaden the CAP of stimulated frog sciatic nerves [8] and restore the CAP of compression injured white matter strips through blockage of potassium channels [35]. To characterize the impact of 4-AP on stimulated CNS nerve fibers, 6 spinal cord white matter strips were separated into two groups: no stimulation or 200 Hz stimulation for 15 min. Afterwards, spinal tissues were incubated with 100 µM 4-AP for 15 min and their CAPs measured following a 1 min wash with Krebs' solution. The CAP waveforms are shown in Fig. 5A–B. Control samples maintained in circulating Krebs' solution without stimulation showed no significant changes in CAP after 4-AP treatment (Fig. 5A). On the other hand, incubation with 4-AP after 200 Hz stimulation resulted in the appearance of a shoulder CAP waveform (Fig. 5B) and an increase in CAP amplitude (Fig. S3). To detect the effect of 200 Hz stimulation and 4-AP on spinal cord's response to multiple stimuli, we measured the refractory period using the method as previously described [36]. Spinal cord tissues were stimulated by dual stimuli with various interval times, ranging from 0.5 to 15 ms. The absolute refractory periods (interval time that second peak starts to appear) for pre-stimulation, post-stimulation and 4-AP treatment were 0.83±0.10, 0.85±0.06, and 0.88±0.05, respectively, with no significant difference (p>0.05). The relative refractory periods (interval time that 2^nd^ peak amplitude is no less than 95% of the 1^st^ peak) were determined to be 5.0±0.0, 6.5±0.6 and 6.5±0.6 for the three groups, with spinal cords post-stimulation and 4-AP treatment significantly higher than the pre-stimulation condition (p<0.01) (Fig. S4).

Utilizing CAP measurement to observe the collective response of many axons to stimulation as well as CARS microscopy to efficiently examine many individual nodes in live spinal tissues, we have explored mechanisms linking repetitive stimulation to paranodal myelin change. An increase in intracellular calcium is known as a key event in the pathology of axons [37], [38]. Lev-Ram and Ellisman observed calcium transients in myelinating Schwann cells of frog sciatic nerve during high-frequency stimulation [39]. It was also noted that action potentials could induce Ca^2+^ influx into mammalian optic nerve stimulated at 25 Hz [40]. To examine the role of Ca^2+^, three white matter strips were maintained in a calcium free Krebs' solution supplemented with 2 mM EGTA for 1 h. Samples were then stimulated at 200 Hz for 15 min followed by a 15 min incubation with 100 mM 4-AP. Subsequent CAP measurement revealed little broadening of the CAP (Fig. 5C) and no increase of CAP amplitude (Fig. S3), supporting that paranodal myelin retraction is a calcium dependent process. A quantitative analysis of the CAP half width at half maximum (HWHM) of these samples (Fig. 5D) demonstrated no significant difference (p>0.1) after either stimulation or 4-AP treatment in constrast to 4-AP rescued samples maintained in normal Krebs' which saw the evolution of a shoulder peak.

**Figure 6:**
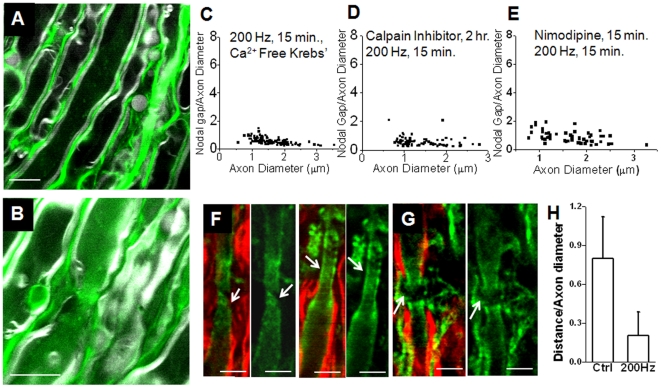
High-frequency stimulation causes Ca^2+^ influx into axons and subsequent activation of calpain. (A–B) CARS imaging of myelin sheath (grey) and TPEF (green) imaging of OG in white matter samples (A) stimulated at 1 Hz for 15 min and (B) stimulated at 200 Hz for 15 min. Calcium indicator can clearly be visualized within axonal space after high frequency stimulation but is only weakly fluorescent in controls. Arrowhead points the node of Ranvier. (C–D) Quantification of myelin retraction reveals incubation in (C) calcium free Krebs' supplemented with 2 mM EGTA reduced retraction to 2±1% (n = 4, 102 nodes total), (D) incubation in calpain inhibitor reduced retraction to 4±1% (n = 3, 81 nodes total) and (E) incubation in nimodipine reduced retraction to 15±7% (n = 4, 78 nodes total). (F) CARS imaging of myelin (red) and TPEF imaging of βII spectrin (green) shows strong labeling of βII spectrin at nodal area, indicated by arrows, in white matter tracts stimulated at 200 Hz. (F) βII spectrin is absent at nodal area in controls stimulated at 1 Hz. (H) Quantification shows that with 200 Hz stimulation, the “bare gap” of βII spectrin at the node reduced to 0.21±0.l8 while control the was 0.80±0.32 (p<0.01). Bar = 5 µm (A), 20 µm (B), and 5 µm (F–G).

To further investigate the potential of calcium influx as an instigator of paranodal myelin retraction, ventral white matter strips were incubated for 1 h in calcium free Krebs' solution supplemented with the calcium indicator Oregon Green BAPTA2-AM (40 µM) prior to high-frequency stimulation. Nodes of Ranvier were examined by simultaneous CARS imaging of myelin sheath (Fig. 6A, grey) and TPEF imaging of OG (Fig. 6A, green). After stimulation, myelin retracted nodes showed a TPEF signal within axonal space 2.5 times greater than samples stimulated at 1 Hz (Fig. 6B). To provide quantitative evidence of the impact of calcium influx, 4 white matter tissues were incubated in Ca^2+^ free Krebs' solution containing 2 mM EGTA. Subsequent 200 Hz resulted in a small percentage (2±1%, p>0.5 vs. 1 Hz stimulation, One-way ANOVA) of myelin retraction in 102 nodes observed (Fig. 6C) indicating that Ca^2+^ is involved in induction of paranodal myelin retraction.

A rapid increase in intracellular calcium could lead to the activation of calcium sensitive enzymes such as calpain, a proteolytic enzyme whose substrates include the cytoskeletal protein spectrin and neurofilaments [41]. Calpain activation could then lead to the breakdown of cytoskeletal proteins [32] thereby disrupting the linkages of myelin anchoring proteins to the cytoskeleton. To investigate the potential role of calpain activation in paranodal myelin retraction, 3 ventral white matter strips were incubated in normal Krebs' supplemented with a calpain inhibitor (Calpain inhibitor III, Sigma-Aldrich, 500 mM) for 1 h prior to stimulation at 200 Hz. Quantification of paranodal myelin retraction (Fig. 6D) revealed that incubation in calpain inhibitor prior to high-frequency stimulation significantly reduced myelin retraction (p<0.01 vs. 200 Hz stimulation without calpain), with 4±1% of nodes examined showing retracted morphology. Nimodipine, an L-type calcium channel blocker, at 10 µM concentration, was applied to the spinal cord tissue 15 min prior to stimulation. Quantification of paranodal myelin retraction (Fig. 6E) revealed that incubation of nimodipine partially reduced myelin retraction (p<0.01), with 15±7% of nodes examined showing retracted morphology. To provide further evidence of calpain activity, immonolabeled βII spectrin was visualized by TPEF imaging in conjunction with F-CARS imaging of myelin sheath. Immunofluorescence mapping of white matter strips stimulated at 200 Hz demonstrated that βII spectrin diffused into nodal area (Fig. 6F) whereas in samples stimulated at 1 Hz the βII spectrin was restricted to paranodal and juxtaparanodal areas (Fig. 6G). The “bare gap” of βII spectrin at the node was reduced to about 1/4 after 200 Hz stimulation (Fig. 6H).

## Discussion

Morphological changes to the paranodal region following periods of high-frequency stimulation have been a topic of interest for understanding axon-myelin interactions. A variety of methods have been employed to investigate whether the structure of the node of Ranvier changes as a result of action potential propagation. X-ray diffraction studies showed enhanced myelin swelling in toad sciatic nerves stimulated at 200 Hz for 1 h prior to perfusion with distilled water [10]. However, the diffraction patterns did not allow for direct visualization of the myelin sheath. Repetitive propagation of action potentials in rat sciatic nerves resulted in both delayed repolarization and increased action potential duration after exposure to a potassium channel blocker [8] which suggested loosening of paranodal myelin. It was later revealed by electron microscopy (EM) that peripheral nerves stimulated at high-frequency showed extensive disruption of the paranodal bulbs [11]. However, the sample fixation and staining procedures necessary for EM precluded real-time observation of the myelin sheath during stimulation.

The current work overcomes the challenges faced by earlier methods by real-time CARS imaging of the response of central myelinated nerve fibers to repetitive action potential propagation. Unlike other methods such as EM or X-ray diffraction, CARS imaging provides high resolution, high-contrast images of the paranodal myelin of individual axons in real-time with minimal sample preparation and no staining, making it possible to follow the dynamic behavior of the paranodal myelin of the same axon throughout the stimulation. As a result, the current study has revealed paranodal myelin retraction during high frequency stimulation of spinal cord white matter. Although previous investigations of paranodal structure in sciatic nerve after high-frequency stimulation did not observe myelin retraction, paranodal myelin vacuolization and slowed conduction velocity were observed, suggesting the exposure of juxtaparanodal potassium channels [11]. That paranodal myelin retraction was not seen is conceivably due to the difference in peripheral versus central nervous system, whereas peripheral nerves are myelinated by Schwann cells and thus possess microvilli and a basal lamina which could provide additional structural support to peripheral myelin, these structures are absent in the CNS.

The current work is the first demonstration of CARS imaging and neuro-physiological measurement on the same platform. This combination allowed us to correlate the collective physiological response of the whole tissue to long periods of repetitive stimulation with structural changes observed at the single nerve fiber level. The CAP measurements and the use of 4-AP provided a better understanding of the observed myelin retraction. After high-frequency stimulation, the CAP amplitude of all samples was decreased, in accordance with conduction failure induced by paranodal myelin retraction and K^+^ channel exposure [42]. Considering the inherent bias of CAP measurements towards large, fast-conducting axons [43], the observed increase in latency and relative refractory period after stimulation was also attributed to the paranodal myelin retraction which was observed to be most prevalent in larger axons (Fig. 3B). Following incubation with 4-AP, the CAP of spinal tissues stimulated at 200 Hz showed a restoration of the CAP peak to pre-stimulation amplitude and the appearance of a shoulder in the waveform, but the sensitivity of 4-AP disappeared after a recovery period of 2 hours (Fig. S5). The broadening of the CAP has previously been observed in a lysophosphatidylcholine induced demyelination model in which axons with paranodal demyelination treated with 4-AP experienced a prolonged action potential due to the blockage of outwards K^+^ current at these sites [44]. Indeed, ventral spinal cord axons in developing rat were found susceptible to CAP broadening due to delayed repolarization following 4-AP treatment [45]. It is also possible that spinal cord axons with myelin retraction display conduction block and that 4-AP restored propagation in slow conducting demyelinated axons. As Rasband and Trimmer found that Kv 1.2 channels are present at paranodes in normal rats [46], the sensitivity of 4-AP to K^+^ channels may not be constrained to juxtaparanodal area but have a larger impact as observed by CAP measurement.

Several possible mechanisms have been proposed for the destabilization of paranodal myelin following high-frequency stimulation. It has been hypothesized that the paranodal disruptions in peripheral nerves could be due to heat generation at the node from repetitive action potentials [11]. However, the initial heat caused by rising phase of the action potential overshooting the repolarization phase is usually small [47]. Another possibility is that the observed structural changes are due to microedema formation in the paranodal bulbs, as excessive potassium efflux could result in increased hypertonicity of periaxonal space [11], but no direct evidence was shown to confirm the contribution of potassium efflux [39].

Our conclusion that paranodal myelin retraction in central myelinated nerve fibers is a calcium dependent process is supported by several observations. First, preincubation in Ca^2+^ free Krebs' solution before 200 Hz stimulation effectively prevented myelin retraction, making these samples statistically indistinguishable from controls (Fig. 6C). Second, incubation of white matter strips with a fluorescent calcium indicator showed clear TPEF signal within axonal space after 200 Hz stimulation (Fig. 6A). Third, incubation with 4-AP did not result in the appearance of a second peak in the CAP of Ca^2+^ free samples, in contrast to the shoulder visible in the CAPs of samples in normal Krebs' (Fig. 5B). Moreover, incubation of white matter samples in a calpain inhibitor prior to stimulation (Fig. 6D) and nimodipine (Fig. 6E) significantly reduced retraction and immunofluorescence imaging of βII spectrin showed diffusive pattern into the nodes in samples stimulated at 200 Hz, suggesting retraction results from cytoskeleton degradation through calpain activity (Fig. 6F). The axonal membrane contains a complex of two cell recognition molecules, contactin-associated protein (Caspr) and contactin, at the axoglial junction [48]. This complex is associated with the glial membrane protein NF 155 whereas the cytoplasmic tail of Caspr is associated with protein 4.1B which contains an actin-spectrin binding sequence and provides a potential anchors to immobilize the Caspr-contactin complex and therefore anchor the paranodal loops to the axon at the nodes of Ranvier [49]. Our results suggest that high-frequency stimulation leads to pathologic influx of extracellular calcium, activating calpain, which in turn degrades the cytoskeletal cross linker, spectrin. Cytoskeletal degradation through spectrin breakdown then leads to paranodal myelin retraction. This mechanism is further supported by the observation that the Kv 1.2 potassium channels at myelin-retracted nodes were no longer sequestered beneath the myelin of the paranode (Fig. 4A,E,F), and NF 155 diffused into juxtaparanodes (Fig. 4C,F). Previous investigations into high-frequency stimulation of peripheral nerve noted Ca^2+^ transients in Schwann cells which triggered release of calcium from intracellular calcium stores [39]. Although calcium influx into myelin was not observed in the present study, calcium influx into myelin or myelinating glia could contribute to the observed paranodal myelin retraction. Similarly, the contribution of elevated local K^+^ concentration beneath the paranode due to repetitive stimulation was not investigated. However, our observation of effective elimination of paranodal myelin retraction by incubation of tissues in a calcium free bath or by treatment with calpain inhibitor support that paranodal myelin retraction is principally a calcium dependent process.

Myelin retraction as a result of repetitive action potential propagation could have some important implications in deep brain stimulation (DBS) and dorsal column stimulation (DCS) of the spinal cord. Targeted stimulation of deep brain structures at frequencies >100 Hz has produced remarkable results in the treatment of essential tremor and Parkinson's disease as well as epilepsy [50] although the mechanism of this therapy is still unclear [51]. Similarly, DCS has provided relief for patients suffering from chronic lower back pain although the underlying mechanisms of DCS are not well defined [6]. Our results show that high-frequency stimulation temporarily compromises the integrity of the paranode of spinal cord axons, leading to exposure of voltage gated potassium channels. Potassium efflux through these channels could contribute to decreased action potentials through conduction failure. Though our observations are made in isolated tissue, the potential to observe many nodes of Ranvier *in vivo* [26] along with the capability to measure the collective response of many axons to stimulation could enable the presented platform as a tool for investigating whether similar effects on nodal structure occur within the stimulation regimes used in DBS or DCS.

In conclusion we have provided direct evidence of myelin retraction at the nodes of Ranvier during high-frequency stimulation by CARS imaging of spinal tissues in real time. Our study revealed that high frequency stimulation leads to pathologic calcium influx which activates the calcium sensitive enzyme calpain inside the axon. The cytoskeletal protein spectrin is broken down by calpain activity, leading to degradation of the cytoskeleton. This degradation results in myelin retraction as the paranodal loops are attached to the cytoskeleton via anchoring proteins. Furthermore, incubation of stimulated spinal tissues with potassium channel blocker 4-AP resulted in the appearance of a second peak in the CAP which was absent in Ca^2+^ free samples. Technically the combination of electrophysiology recordings with high speed CARS imaging of myelin sheath without labeling provides a new platform for the investigation of myelin function in the nervous system.

## Supporting Information

Figure S1 The localization of Kv 1.2 before stimulation, after stimulation and after a two hour recovery period. The ratio was calculated as the distance between Kv 1.2 on each side of the node normalized by axon diameter. After 200 Hz stimulation, the distance ratio was reduced to 0.59±0.14 from 2.15±0.25. After a two-hour recovery period, the ratio was increased to 1.19±0.18.
(TIF)

Figure S2 The junction protein Caspr relocated further away from the paranodal area and diffused along the axon. We characterized the location of Caspr by measuring the gap distance between Caspr at each side of the node normalized by the axon diameter. The nodal gap ratio was increased to 1.49±0.17 in 200 Hz stimulated spinal cords as compared with 0.53±0.05 in 1 Hz control (*p*>0.01). We also characterized the length of Caspr by measuring the length of the fluorescence labels normalized by the axon diameter. The Caspr length ratio was increased to 5.64±0.94 in 200 Hz stimulated spinal cords while the control showed 3.97±0.32 (*p*>0.01).
(TIF)

Figure S3 CAP amplitudes after 200 Hz stimulation and 4-AP treatment. At the presence of calcium, CAP amplitude decreased to 91.9±5.1 post stimulation, and increased to 97.0±5.0 following 4-AP treatment (p<0.01). While without the presence of calcium, CAP amplitude maintained at 100.6±6.4 post stimulation and 99.4±3.2 after 4-AP treatment (p>0.05).
(TIF)

Figure S4 The refractory period measurement. Spinal cord tissue was stimulated by dual stimuli with various interval times, ranging from 0.5 to 15 ms. The absolute refractory periods (interval time that second peak starts to appear) for pre-stimulation, post-stimulation and 4-AP treatment were 0.83±0.10, 0.85±0.06, and 0.88±0.05, respectively, with no significant difference (p>0.05). The relative refractory periods (interval time that 2^nd^ peak amplitude is no less than 95% of the 1^st^ peak) were determined to be 5.0±0.0, 6.5±0.6 and 6.5±0.6 for the three groups, with spinal cords post-stimulation and 4-AP treatment significantly higher than the pre-stimulation condition (p<0.01).
(TIF)

Figure S5 CAP amplitudes after 200 Hz stimulation and 4-AP treatment after a two-hour recovery period. CAP amplitude decreased to 92.8±24.1 following 4-AP treatment without significant difference compared with initial and post stimulation conditions (p>0.05).
(TIF)

Movie S1 Real-time CARS imaging of paranodal myelin retraction in response to 200 Hz stimulation for 20 min.
(AVI)
