# Supplementary figures and images for: Real-Time CARS Imaging Reveals a Calpain-Dependent Pathway for Paranodal Myelin Retraction during High-Frequency Stimulation
Source: PLoS One. 2011 Mar 3;6(3):e17176. doi: 10.1371/journal.pone.0017176 (PMC3048389; doi:10.1371/journal.pone.0017176)

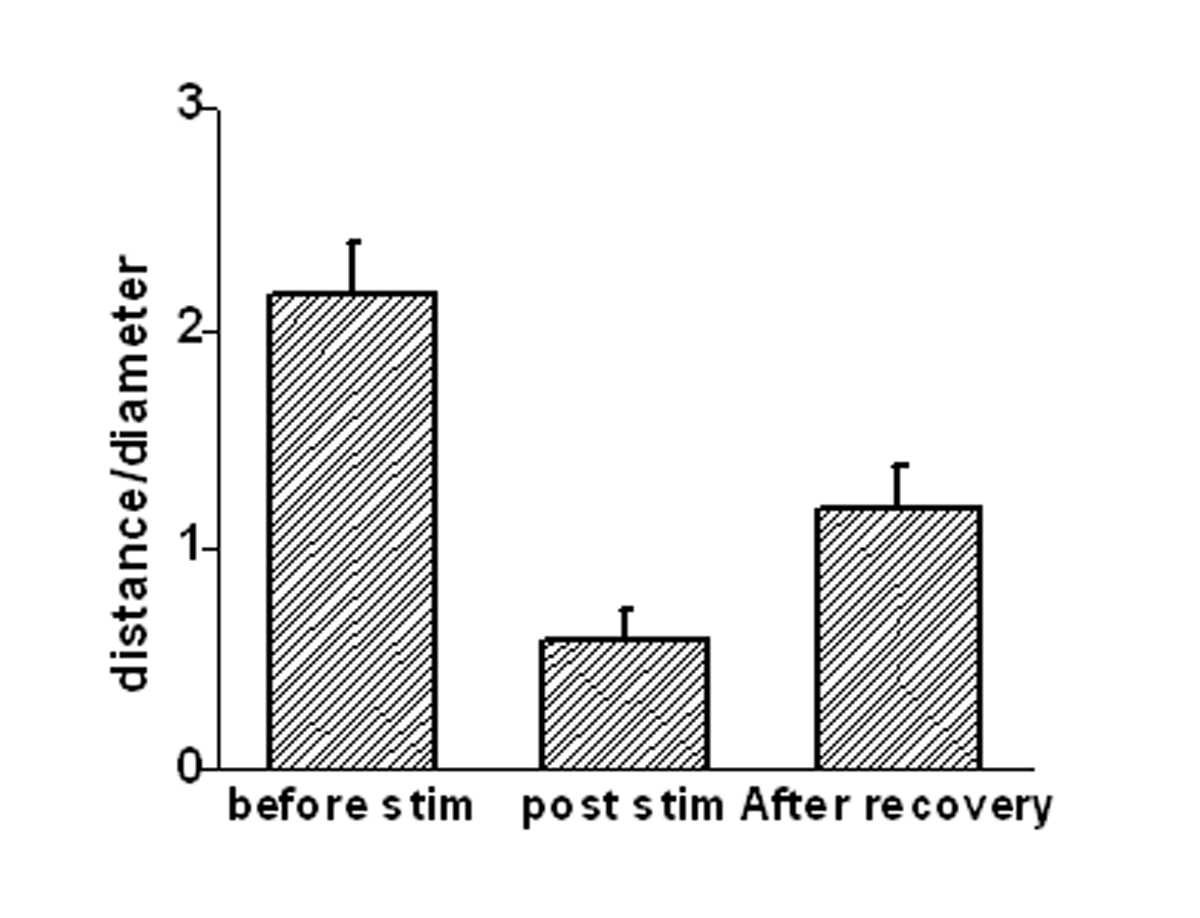

Supplement: Figure S1 — The localization of Kv 1.2 before stimulation, after stimulation and after a two hour recovery period. The ratio was calculated as the distance between Kv 1.2 on each side of the node normalized by axon diameter. After 200 Hz stimulation, the distance ratio was reduced to 0.59±0.14 from 2.15±0.25. After a two-hour recovery period, the ratio was increased to 1.19±0.18. (TIF) [file pone.0017176.s001.tif]

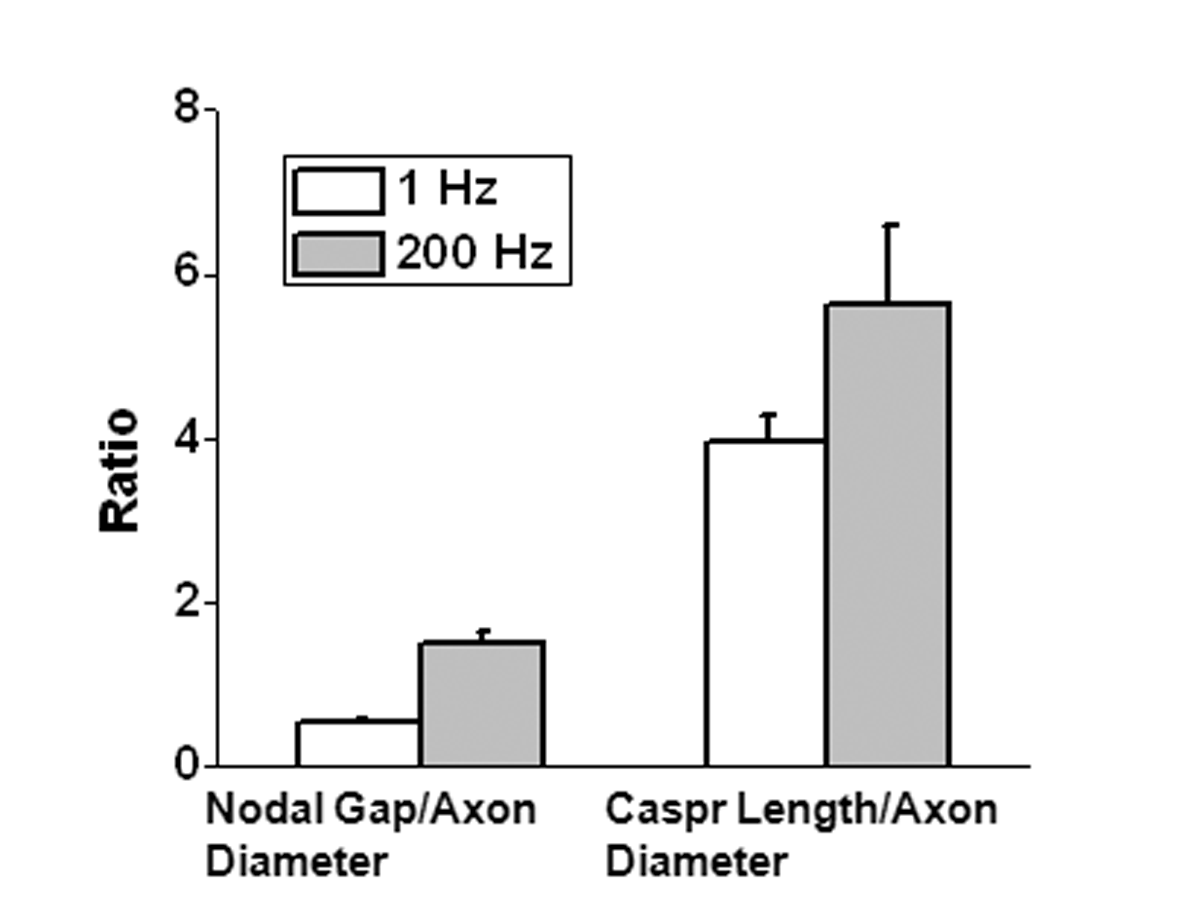

Supplement: Figure S2 — The junction protein Caspr relocated further away from the paranodal area and diffused along the axon. We characterized the location of Caspr by measuring the gap distance between Caspr at each side of the node normalized by the axon diameter. The nodal gap ratio was increased to 1.49±0.17 in 200 Hz stimulated spinal cords as compared with 0.53±0.05 in 1 Hz control (p>0.01). We also characterized the length of Caspr by measuring the length of the fluorescence labels normalized by the axon diameter. The Caspr length ratio was increased to 5.64±0.94 in 200 Hz stimulated spinal cords while the control showed 3.97±0.32 (p>0.01). (TIF) [file pone.0017176.s002.tif]

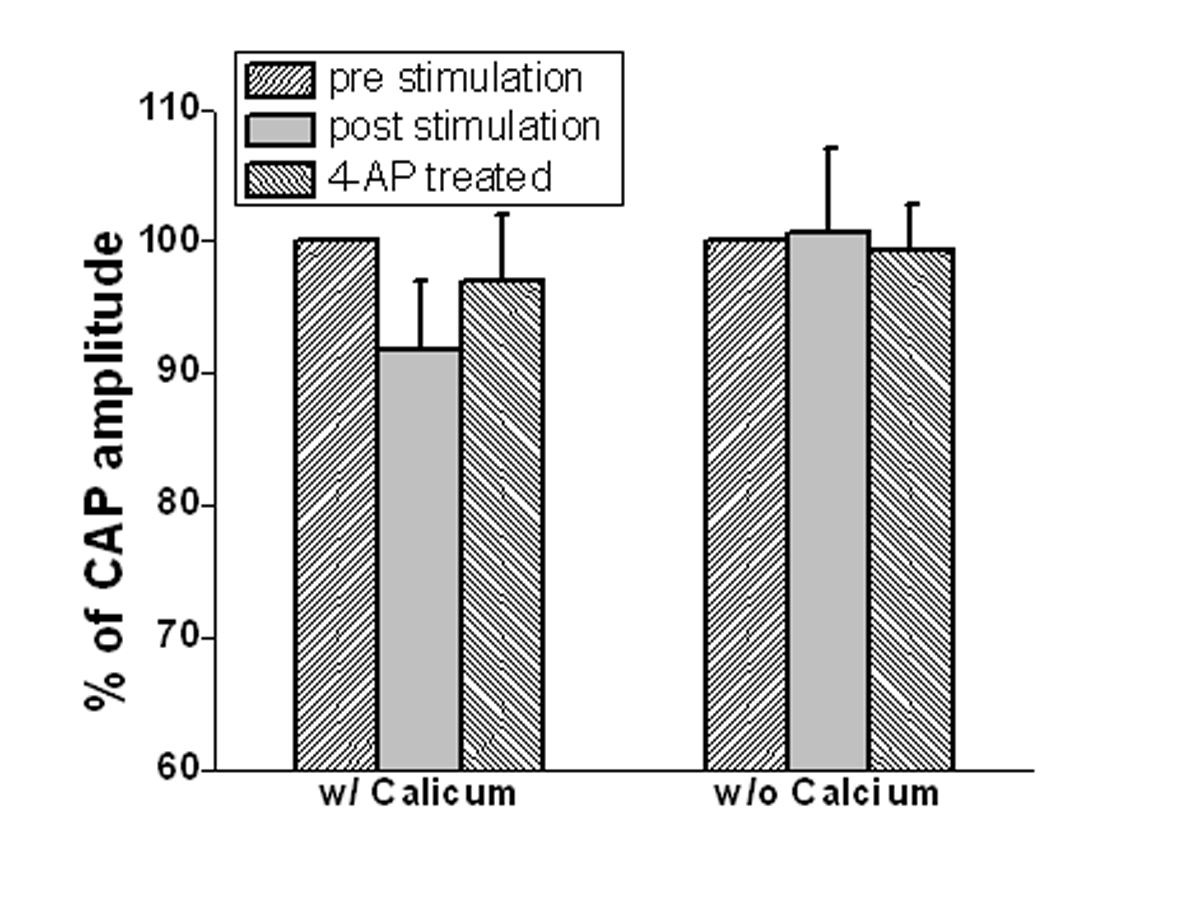

Supplement: Figure S3 — CAP amplitudes after 200 Hz stimulation and 4-AP treatment. At the presence of calcium, CAP amplitude decreased to 91.9±5.1 post stimulation, and increased to 97.0±5.0 following 4-AP treatment (p<0.01). While without the presence of calcium, CAP amplitude maintained at 100.6±6.4 post stimulation and 99.4±3.2 after 4-AP treatment (p>0.05). (TIF) [file pone.0017176.s003.tif]

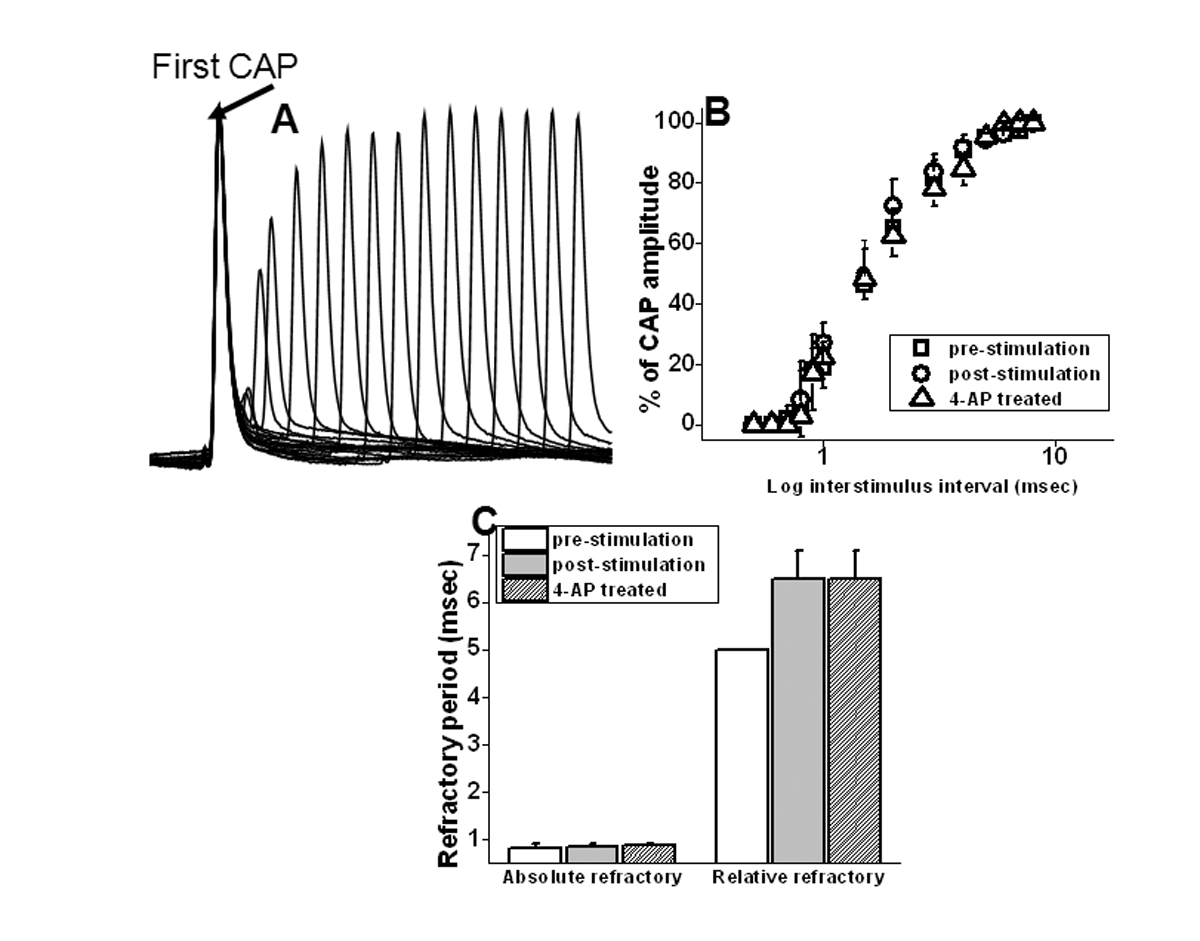

Supplement: Figure S4 — The refractory period measurement. Spinal cord tissue was stimulated by dual stimuli with various interval times, ranging from 0.5 to 15 ms. The absolute refractory periods (interval time that second peak starts to appear) for pre-stimulation, post-stimulation and 4-AP treatment were 0.83±0.10, 0.85±0.06, and 0.88±0.05, respectively, with no significant difference (p>0.05). The relative refractory periods (interval time that 2nd peak amplitude is no less than 95% of the 1st peak) were determined to be 5.0±0.0, 6.5±0.6 and 6.5±0.6 for the three groups, with spinal cords post-stimulation and 4-AP treatment significantly higher than the pre-stimulation condition (p<0.01). (TIF) [file pone.0017176.s004.tif]

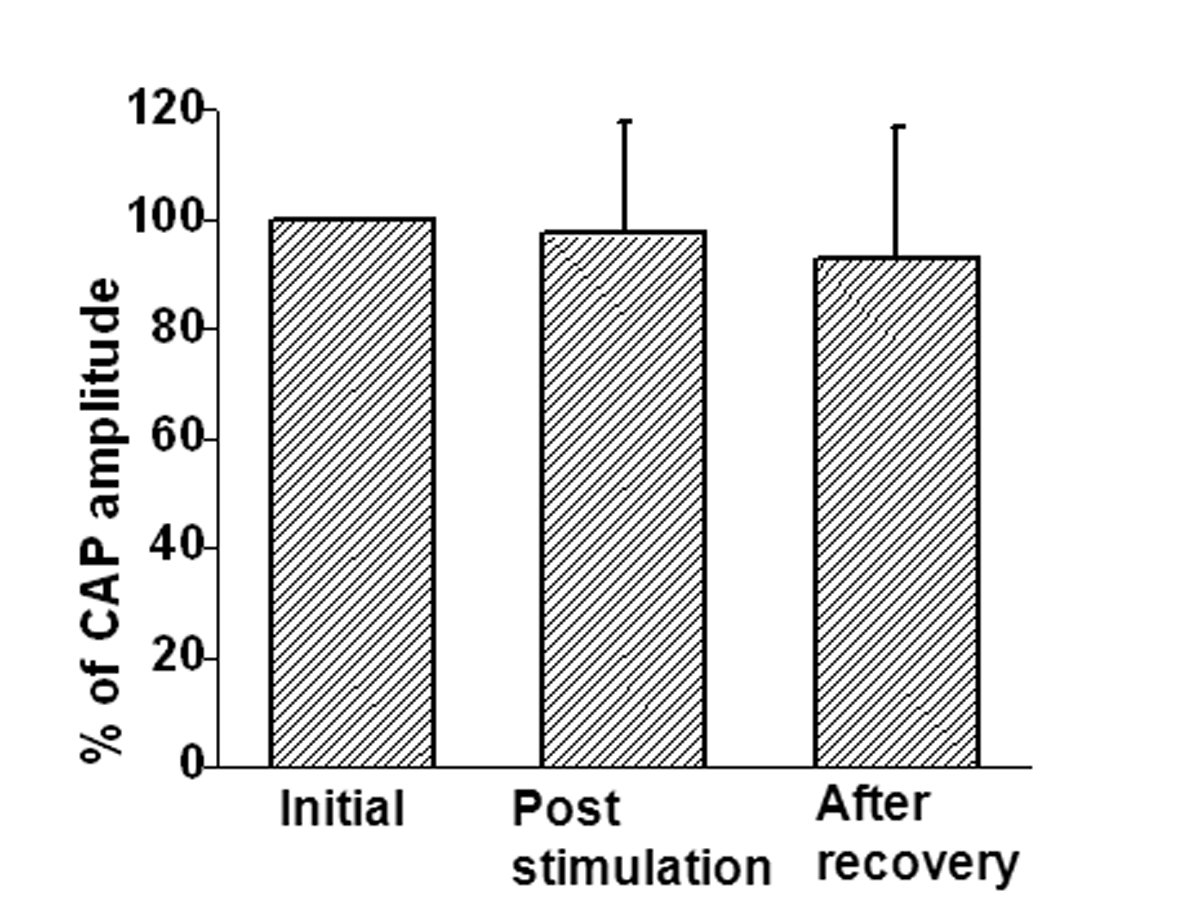

Supplement: Figure S5 — CAP amplitudes after 200 Hz stimulation and 4-AP treatment after a two-hour recovery period. CAP amplitude decreased to 92.8±24.1 following 4-AP treatment without significant difference compared with initial and post stimulation conditions (p>0.05). (TIF) [file pone.0017176.s005.tif]
